# Complete mitochondrial genome of *Casmara patrona* (Lepidoptera: Oecophoridae)

**DOI:** 10.1080/23802359.2020.1863872

**Published:** 2021-02-05

**Authors:** Kai Jiang, Tianjuan Su, Bo He, Fang Zhao, Gonghua Lin, Zuhao Huang

**Affiliations:** School of Life Sciences, Jinggangshan University, Ji’an, China

**Keywords:** *Casmara patrona*, Oecophoridae, mitogenome, phylogenetics

## Abstract

The complete mitochondrial genome of *Casmara patrona* (Lepidoptera: Oecophoridae) was sequenced for a future phylogenetic study of Lepidoptera. The circle genome of the moth is 15,393 bp in length with a pronounced base bias of A + T (79.3%), containing 13 protein-coding genes, 22 transfer RNAs, two ribosomal RNAs, and a putative control region. The *coxI* gene had a CGA start codon as most lepidopteran species, other PCGs use the typical ATN codons. All PCGs end with the complete stop codon TAA. Phylogenetic analyses showed that the monophyly of Oecophoridae was highly supported based on the concatenated sequence of the 13 PCGs. In addition, Oecophoridae and Xyloryctidae had the closest relationship.

Gelechioidea is the second most species-rich group of Lepidoptera that is important to attaining a better understanding of higher-level phylogeny (Kaila et al. 2011; Nieukerken et al. 2011). It is distributed worldwide, comprising of 18,489 species in 1428 genera (Nieukerken et al. 2011). Oecophoridae is one of the most species-rich Gelechioidea family with worldwide distribution, which comprises more than 3000 described species and numerous undescribed ones (Nieukerken et al. 2011). *Casmara patrona*, belonging to Lepidoptera, Oecophoridae, which distribution range is similar to that of *Camellia oleifera*. It is reported as the economically important pest of *C. oleifera*, which is one of the main borer pests damaging *C. oleifera* in China. Few reports about this moth were published. In this study, we present the complete mitogenome of *C. patrona* (Lepidoptera: Oecophoridae) for a future phylogenetic study of Lepidoptera.

The *C. patrona* specimen was collected from Ji’an, Jiangxi Province, China (27.10577, 115.01747) in August 2019, and were stored in a −20 °C refrigerator that was preserved at Entomological Specimen Room of Jinggangshan University (accession number: 20190815S). Total genomic DNA was extracted from a single specimen with the QIAamp DNA Mini Kit (QIAGEN, Hilden, Germany) following the manufacturer’s instruction. A total of 1.5 μg whole genomic DNA was sequenced by Illumina HiSeq 4000 platform (Illumina, San Diego, CA), with a read length of 150 bp. The reads were used for mitogenome *de novo* assembly with MEGAHIT v1.1.2 (Li et al. 2016) and the complete mitogenome was annotated using the MITOS web server (Bernt et al. 2013).

The complete mitogenome of *C. patrona* (GenBank accession no. MW006609) is a circular DNA of 15,393 bp in length. Similar to most insect mitogenomes, it contains the typical set of 37 genes (Cameron 2014), which include 13 protein-coding genes (PCGs), two ribosomal RNA genes (*rrnL* and *rrnS*), 22 transfer RNA (tRNA) genes, and an AT rich control region (D-loop). The heavy strand encodes nine PCGs and 14 tRNAs, while four PCGs, eight tRNAs, and two rRNAs are located on the light strand. The overall base composition of *C. patrona* mitogenome is 40.4% T, 38.8% A, 13.3% C, and 7.5% G, which with a pronounced base bias of A + T (79.2%). Like the majority of other lepidopterans, it is characterized by its remarkably high A + T content (Wang et al. 2016). Twelve of the 13 PCGs started with ATN codons (ATT and ATG); however, the COI gene began with CGA (arginine). All PCGs use the common stop codon TAA. The *rrnL* is located between *trn-Leu1* and *trn-Val*, with the length of 1292 bp. The *rrnS* is located between *trn-Val* and the control region, with the length of 784 bp. The AT rich control region is 336 bp in length, which is located between *rrnS* and *trn-Met*.

The nucleotide sequences of the 13 PCGs of eight Gelechioidea species and four outgroup species were concatenated for phylogenetic analysis (Figure 1). The best-fit partitioning scheme and nucleotide substitution model (GTR + I + G) for tree reconstruction was determined by the Akaike information criterion implemented in jModelTest v2.1.6 (Darriba et al. 2012). The program MrBayes v3.2.6 (Ronquist 2012) was used to reconstruct phylogenetic inference trees with 10,00,000 generations, which are sufficient to meet the 0.01 criterion of the standard deviation of split frequencies. As expected, all species assembled into a monophyletic clade within each subgenus. Within the Gelechioidea, *C. patrona* was grouped with *Endrosis sarcitrella* as the sister group (Figure 1). In addition, the monophyly of Oecophoridae was highly supported (Figure 1). Our results emphasize the value of *C. patrona* mitogenome to phylogenetic analyses of moths.

**Figure 1.**
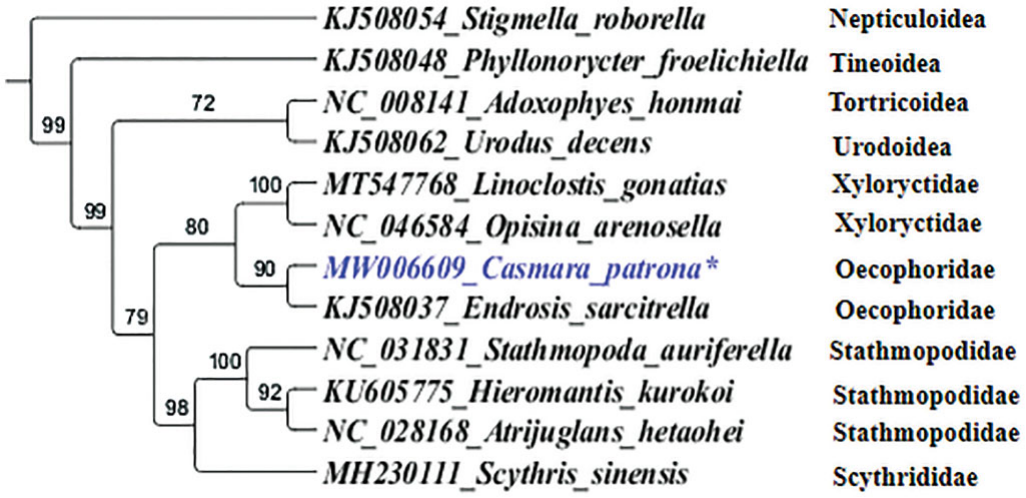
Phylogenetic relationship of Gelechioidea based on the BI analyses of the concatenated nucleotide sequences of 13 PCGs. The stars indicate the focal mitochondrial genomes in this study. Species names are followed by the GenBank accession numbers of their mitochondrial genomes.

## Data Availability

The data that support the findings of this study are openly available in GenBank of NCBI at https://www.ncbi.nlm.nih.gov, reference number MW006609.
